# Enhanced Interleukin 6 Trans-Signaling Modulates Disease Process in Amyotrophic Lateral Sclerosis Mouse Models

**DOI:** 10.3390/brainsci15010084

**Published:** 2025-01-17

**Authors:** Carol Milligan, Dale O. Cowley, William Stewart, Alyson M. Curry, Elizabeth Forbes, Brian Rector, Annette Hastie, Liang Liu, Gregory A. Hawkins

**Affiliations:** 1 Department of Translational Neuroscience, Wake Forest University School of Medicine, Winston-Salem, NC 27157, USA; 2 Department of Genetics and Animal Models Core Facility, University of North Carolina, Chapel Hill, NC 27599, USA; dale_cowley@med.unc.edu; 3 Department of Cancer Biology, Wake Forest University School of Medicine, Winston-Salem, NC 27157, USA; 4 Department of Internal Medicine Section on Pulmonary, Critical Care, Allergy and Immunologic Diseases, Wake Forest University School of Medicine, Winston-Salem, NC 27157, USA; 5 Department of Biochemistry, Wake Forest University School of Medicine, Winston-Salem, NC 27157, USA

**Keywords:** motor neuron disease, neuroimmune, glial cells, neuromuscular junctions, microglia, astrocytes, motoneurons

## Abstract

**Background/Objectives:** Charcot first described ALS in 1869, but the specific mechanisms that mediate the disease pathology are still not clear. Intense research efforts have provided insight into unique neuroanatomical regions, specific neuronal populations and genetic associations for ALS and other neurodegenerative diseases; however, the experimental results also suggest a convergence of these events to common toxic pathways. We propose that common toxic pathways can be therapeutically targeted, and this intervention will be effective in slowing progression and improving patient quality of life. Here, we focus on understanding the role of IL6 trans-signaling in ALS disease processes. **Methods:** We leveraged unique mouse models of IL6 trans-signaling that we developed that recapitulate the production of active sIL6R in a genotypic and quantitative fashion observed in humans. Given that the SOD1 transgenic mouse is one of the most highly studied and characterized models of ALS, we bred *SOD1^G93A^* mice with IL6R trans-signaling mice to determine how enhanced trans-signaling influenced symptom onset and pathological processes, including neuromuscular junction (NMJ) denervation, glial activation and motoneuron (MN) survival. **Results:** The results indicate that in animals with enhanced trans-signaling, symptom onset and pathological processes were accelerated, suggesting a role in disease modification. Administration of an IL6R functional blocking antibody failed to alter accelerated symptom onset and disease progression. **Conclusions:** Future work to investigate the site-specific influence of enhanced IL6 trans-signaling and the tissue-specific bioavailability of potential therapeutics will be necessary to identify targets for precise therapeutic interventions that may limit disease progression in the 60% of ALS patients who inherit the common *Il6R* Asp^358^Ala variant.

## 1. Introduction

Regardless of family history, site of onset and sequence of symptoms and progression, the majority of amyotrophic latera sclerosis (ALS) patients die within 2–5 years of diagnosis. Truly effective treatments for neurodegenerative diseases such as ALS are elusive. Recent genetic approaches promoting the expression of the spinal motor neuron (SMN) protein in spinal muscular atrophies (SMAs) such as nuinersen have provided life-changing effects for patients and their families. This and other SMA therapies were the result of the identification of the genetic cause of the disease and decades of research into the development of approaches and technology to effectively target that cause. For ALS patients with genetically inherited forms of the disease, effective treatments to slow or even “cure” the disease may be on the distant horizon, as new drug delivery technologies are now allowing for gene-targeting approaches to be investigated. But for the majority of ALS patients without a clear genetic disposition, there is much work to do to understand the causes of sporadic disease. The current investigation focuses on a genetically inheritable modifier of ALS and the resulting pathological responses that may be amenable to therapeutic interventions. Further, as many of the pathological responses appear to be shared across neurodegenerative diseases, these approaches could have a more broad impact on other neurodegenerative diseases such as Alzheimer’s disease (AD).

Interleukin 6 (IL6) signaling is an intriguing target to study in neurodegenerative diseases, where non-canonical IL6 trans-signaling is thought to be the dominant pathological mechanism in the CNS [1]. In humans, non-canonical IL6 trans-signaling is driven by inheriting the *Il6R* Asp^358^Ala mutation, which results in a significant increase in IL6 receptor shedding and the production of soluble IL6 receptor (sIL6R) [2,3,4]. Inheritance of the common *Il6R* Asp^358^Ala variant (s2228145; C allele) accounts for the specific elevation of IL6 signaling via GP130 in ALS patients in both serum and cerebral spinal fluid (CSF), with these individuals predisposed to a more rapid progression [5,6]. We have reported that the *Il6R* Asp^358^Ala variant may be an important biomarker of cognitive decline and associates with pathological indicators of AD in individuals with mild cognitive impairment [7]. Importantly, pathological consequences of significant increases in IL6 trans-signaling occur in 60% of ALS and AD patients. Finally, in a clinical trial of tocilizumab, the IL6 trans-signaling blocking drug, ALS patients who received the treatment exhibited a significant reduction in plasma levels of C-reactive protein (CRP), indicating functional inhibition of IL6 signaling. However, only individuals who inherited the *Il6R* Ala^358^ allele exhibited reductions in CSF CRP, suggesting a unique and potentially disease-promoting, CNS IL6-signaling inflammatory response [8]. Taken together, these results raise important questions regarding the role of IL6 trans-signaling as a distinct process that contributes to accelerating disease pathologies in neurodegenerative diseases such as ALS. In this study, we leveraged unique mouse models of IL6 trans-signaling that we developed that recapitulate the production of active sIL6R in a genotypic and quantitative fashion observed in humans. Given that the SOD1 transgenic mouse is one of the most highly studied and characterized models of ALS, we crossed *SOD1^G93A^* mice with IL6R trans-signaling mice to determine how enhanced trans-signaling influences motor weakness and pathological processes, including neuromuscular junction (NMJ) denervation, glial activation and motoneuron (MN) survival.

## 2. Materials and Methods

### 2.1. Mouse Models

All animal experiments conformed to National Institutes of Health guidelines and were approved by the Wake Forest University Animal Care and Use Committee.

### 2.2. Creation and Characterization of IL6 Trans-Signaling Mouse Models

**Figure 1:**
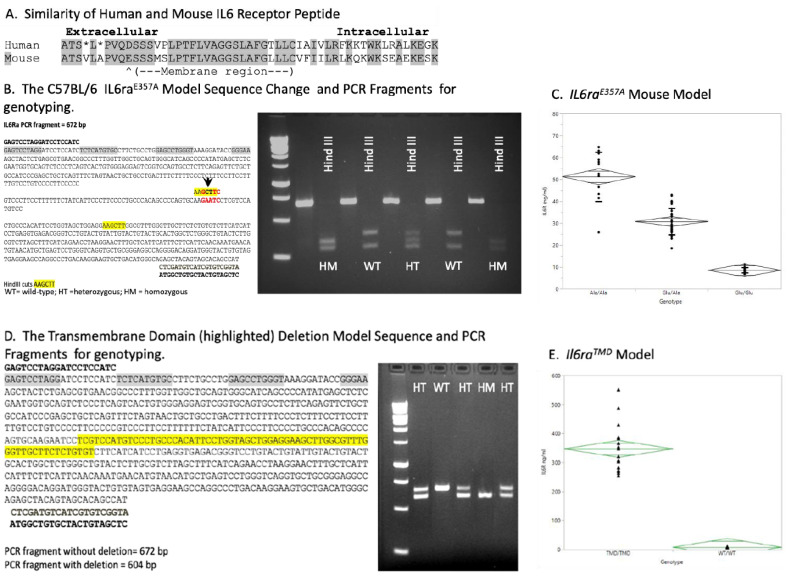

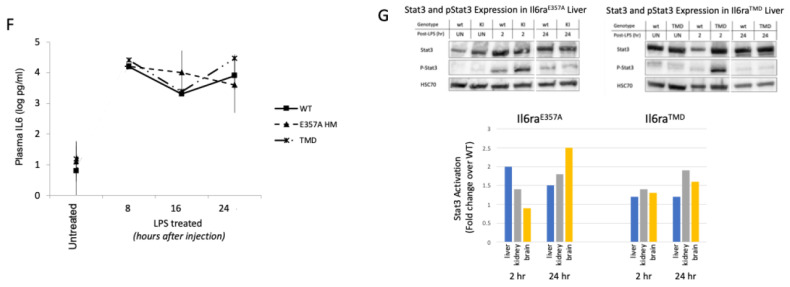
Generation of unique knock-in mouse model C57BL/6 *Il6ra*^E357A^ and IL6R transmembrane deletion (TMD) mouse models. To accurately investigate the potential mechanisms by which IL6 trans-signaling contributes to disease progression, it was necessary to generate a knock-in mouse model C57BL/6 *Il6ra*^E357A^ of IL6 trans-signaling. The mouse *Il6ra* gene was altered by incorporating a two-base-pair change (AA > CT) at the codon for amino acid 357, thus converting the Glu^357^ (GAA) to Ala^357^ (GCT) (**A**,**B**). Incorporating this codon change also produced a novel Hind III site that allows us to identify mice heterozygous (HT) or homozygous (HM) for the E^357^A allele (**B**). ELISA measurement of soluble IL6 receptor in (**C**) *Il6ra^E357A^* mice at P90 (Ala/Ala n = 13; Ala/Glu n = 35; Glu/Glu n = 19; *p* < 0.001 across genotypes; one-way ANOVA) confirmed increased concentrations of soluble receptor in serum. We also created a unique IL6R transmembrane deletion (TMD) mouse model that exhibits tremendous shedding of the receptor (**D**). ELISA measurements of soluble IL6 receptor in *Il6ra*^TMD^ mice at P90 (TMD/TMD n = 17; WT/WT n = 16; *p* < 0.001 across genotypes; one-way ANOVA) are plotted (**E**). ELISA measurements of plasma IL6 in untreated or LPS-treated (3 ug/g, i.p) WT, E^357^A homozygous and TMD homozygous mice at indicated time points (**F**). For each treatment group, P90 sex-matched, littermate WT and Il6ra littermates were used. Littermate, gender-matched animals were used (n = 2–3 groups/treatment group/time point). (**G**) Shown are representative Western blots of untreated or LPS-treated WT, E357A homozygous (HM) and TMD homozygous liver protein extracts 2 and 24 h after LPS administration. Littermate, gender-matched animals were used per treatment group/time point (liver, brain n = 3; kidney n = 2). Phosphorylated Stat3 and total Stat3 levels were normalized to Hsc70 used as a loading control and the ratio of P-Stat3/total Stat3 determined for each tissue sample. Results are expressed as fold-change in *Il6ra* over WT littermate. Phosphorylated Stat3 expression increased by 2 h in all animals but was greater in the *il6ra* models, a pattern observed at 24 h.

Two approaches were utilized to create mouse models of enhanced IL6 trans-signaling using CRISPR/Cas9 technology on the C57BL/6 background. The first approach generated a knock-in mouse model of IL6 trans-signaling, C57BL/6-*Il6ra*^E357A^. The mouse *Il6ra* gene was altered by incorporating a two-base-pair change (AA > CT) at the codon for amino acid 357, thus converting the Glu^357^ (GAA) to Ala^357^ (GCT). This Glu^357^ to Ala^357^ change was located in the mouse *Il6ra* at the reciprocal site of the human Asp^358^Ala change (Figure 1A). In the second approach, a unique IL6Ra transmembrane deletion (TMD) mouse model C57BL/6-*Il6ra^TMD^* was created. Our strategy was to create an in-frame deletion of the membrane-spanning domain, encompassing amino acids S359 to C380 in the mouse *Il6ra* gene. Eliminating the transmembrane portion of the mouse IL6Ra protein should promote secretion into the extracellular space, as reported for an alternatively spliced human IL6RA isoform [9]. In homozygous TMD mice, there is no membrane-bound receptor, and all responses to IL6 are through trans-signaling.

### 2.3. Guide RNA Production and Testing

Multiple CRISPR/Cas9 guide RNAs targeting the coding region for the Glu^357^ codon and membrane-spanning domain were identified using Benchling software “https://benchling.com (accessed on 15 June 2018)”. Five potential guide RNAs with low predicted off-target activity and high predicted on-target activity were produced for testing. Protospacer sequences were cloned into a T7-promoter single-guide RNA scaffold vector (UNC Animal Models Core) followed by T7 in vitro transcription (HiScribe T7 High-Yield RNA Synthesis Kit, New England BioLabs, Ipswich, MA, USA) and RNeasy spin column purification (Qiagen, Germantown, MD, USA), with elution in microinjection buffer (5 mM tris-HCl pH 7.5, 0.1 mM EDTA). Guide RNAs were validated in vitro by incubating guide RNA, recombinant Cas9 enzyme (UNC Animal Models Core/UNC Protein Expression Core Facility) and the PCR-amplified target site, followed by gel electrophoresis to determine the extent of in vitro cleavage activity. Guide RNAs were also validated by the transfection of Cas9/guide RNA complexes in a mouse embryonic fibroblast cell line followed by a T7 endonuclease I assay to detect Cas9/guide RNA activity. Two active guide RNAs were selected for embryo injections to produce the mouse models. Guide RNA Il6ra-sg75B (5′-gacgaggattcttgcact-3′) overlaps the Glu^357^ codon and guide RNA Il6ra-sg69T (5′-gaggaagcttggcgttt-3′) targets near the 3′ end of the *Il6ra* transmembrane domain.

### 2.4. Embryo Electroporation and Microinjection

C57BL/6J females were superovulated by injection with pregnant mare’s serum gonadotropin (PMSG) and human chorionic gonadotropin (HCG) and then mated with C57BL/6J males for zygote production. One-cell embryos were collected from the ampulla oviducts the morning after mating. For the C57BL/6-*Il6ra*^E357A^ model, embryos were electroporated with 1.2 μM recombinant Cas9 protein, 47 ng/μL Il6ra-sg75B guide RNA and 400 ng/μL Il6ra-E357A-B donor oligonucleotide (5′-CAAACGCCAAGCTTCCTCCAGCTACCAGGAATGTGGGCAGGGACATGGACGAGGAAGCTTGCACTGGGGCTGTGGGCAGGGGAAGGGAATGATAGAAAAAAGGAAGGGAC-3′) in Opti-MEM (Gibco). For the C57BL/6-*Il6ra^TMD^* model, embryos were microinjected with 400 nM recombinant Cas9 protein, 25 ng/μL each Il6ra-sg75B and Il6ra-sg69T guide RNAs and 400 ng/μL Il6ra-TMD-B donor oligonucleotide (5′-CAGTGCAGTACAGTACAATACAGTACAGGACCCGTCTCACCTCAGGATGATGAAGGGATTCTTGCACTGGGGCTGTGGGCAGGGGAAGGGAATGATAGAAAAAAGGAAGG-3′) in microinjection buffer. The electroporated or microinjected embryos were implanted into B6D2 pseudopregnant recipients. The resulting pups were screened by sequencing PCR products amplified from the target region. One or more founders with the desired mutation were then mated back to wild-type C57BL/6J animals for germline transmission of the mutant allele.

### 2.5. Genotyping and Initial Characterization

To genotype the C57BL/6-*Il6ra*^E357A^ mice, primers were designed to span the transmembrane domain and site of metalloproteinase cleavage of the IL6 receptor from the cell surface [2,3,4,10]. The forward primer was GAGTCCTAGGATCCTCCATC (Integrated DNA Technologies, 450075339, Coralville, Iowa, USA), and the reverse primer was ATGGCTGTGCTACTGTAGCTC (Integrated DNA Technologies, 450075340, Coralville, Iowa, USA). For genotyping, DNA was obtained from a tail snip and alkaline lysis extraction [11]. An amount of 22.5 ng was used in each polymerase chain reaction (PCR) sample, which were prepared according to the manufacturer’s recommendations (Promega PCR master mix; PRM7502, Coralville, Iowa, USA). The PCR reaction was as follows: 35 cycles of 94 °C for 30 s to denature, 60 °C for 30 s to anneal, 72 °C for 45 s to extend; one cycle of 72 °C for 7 min, store at 4 °C. The Glu^357^ to Ala^357^ change in *Il6ra* was validated by Sanger sequencing. The Glu^357^ to Ala^357^ change was engineered to introduce a novel Hind III site that would be present in the *Il6ra^E357A^* animal PCR product to facilitate genotyping. Digestion of the PCR product (half of the original PCR reaction sample) with Hind III according to the manufacturer’s recommendation (Promega R6048) allows the identification of mice heterozygous (HT) or homozygous (HM) for the E^357^A allele. The PCR and Hind III digestion products were run on agarose gels and stained with ethidium bromide (Figure 1B). Soluble Il6Ra levels were determined in serum using commercially available ELISA assays (R&D Systems mouse IL6Ra ELISA #MR600, Minneapolis, MN, USA; Figure 1C).

The same primer set and PCR reaction designed for *Il6raE^357^A* was used to amplify *Il6ra* in the TMD mice (see Figure 1D). The TMD change in *Il6ra* was validated by Sanger sequencing. Soluble Il6Ra levels were determined in serum as previously described above (Figure 1E).

LPS is a well-studied and potent systemic inducer of inflammation and immune responses that include IL6 [12,13,14,15]. To determine the potential biological effects of enhanced IL6 trans-signaling caused by the availability of increased levels of soluble IL6R in the transgenic mouse models, we administered lipopolysaccharide (LPS) (Sigma; 3 μg/g in sterile saline) via intraperitoneal injection. At selected time points, plasma was collected to measure levels of IL6 in LPS-treated and untreated animals using a commercially available ELISA assay (R&D Mouse IL6 DuoSet Kit DY406-05, Minneapolis, MN, USA). Reports indicate that increased levels of *Il6ra* mRNA are observed in mice within 2–6 h of i.p. LPS injection [12,13], and we determined IL6 plasma levels are increased by 8 h and maintained for at least 24 h (Figure 1F).

The signal transducer and activator of transcription 3 (Stat3) is a key transcription factor that is phosphorylated during activation by IL6 signaling [16,17]. Western blot analysis was performed to determine if there was enhanced and sustained activation/phosphorylation of Stat3 following lipopolysaccharide (LPS) treatments in the liver, brain and kidney. The liver, brain and kidney were also collected and protein extracts isolated in RIPA buffer with protease and phosphatase inhibitors (Cell Signaling Protease/Phosphatase Inhibitor Cocktail 100X #5872s, Danvers, MA, USA). Protein concentrations were determined using the Pierce BCA Assay (ThermoFisher, Waltham, MA, USA) and proteins separated on 12% SDS-PAGE gel. Proteins were transferred to Immobilon-FL PVDF membranes (Millipore, Burlington, MA, USA). Membranes were blocked with 3% BSA in tris-buffered saline (TBS) with 0.05% Tween 20. The membrane was incubated with primary antibody overnight at 4 °C, rinsed in TBST and incubated with the secondary antibodies diluted in 3% BSA in TBST at room temperature for 1 h. Phosphorylated Stat3 (1:1000, rabbit Phospho-Stat3 (Tyr705) Antibody #9131, Cell Signaling Technology, Danvers, MA, USA) and total Stat3 (1:1000, Stat3 (124H6) mouse Mab #9139, Cell Signaling Technology, Danvers, MA, USA) levels were normalized to Hsc70 (1:20,000, Rt Mab #IB5, Enzo Biochem, Farmingdale, NY, USA) and the ratio of P-Stat3/total Stat3 determined for each tissue sample. The secondary antibodies used were IRDye 800CW anti-mouse (1:5000, LI-COR, Lincoln, NE, USA #926-32212), Alexa 488 anti-rat (1:5000, Invitrogen, Waltham, MA, USA #A-21208) or IRDye 680RD anti-rabbit (1:5000, LI-COR, #926-68073). Images were acquired using the ChemiDoc Imaging System (Bio-Rad, Hercules, CA, USA), and then the intensity of the bands was analyzed with Bio-Rad Image Lab software (version 6.1). Bands were quantified using adjusted volume. Stat3 activation was then determined as P-Stat3/Stat3. The results are presented as the mean ± SEM expressed as the fold-change in Stat 3 activation in each IL6Ra over WT littermate control pair. Stat3 phosphorylation in the liver, a tissue with high levels of expression of IL6ra, is elevated at two hours following LPS administration in both models. While this activation subsides by 24 h, tissues with low levels of IL6R expression (e.g., the brain and kidney) show sustained Stat3 activation in LPS-treated *Il6ra*^E357A^ and *Il6ra*^TMD^ homozygous mice as compared to WT littermates (Figure 1G). Our characterization of the *Il6ra* models revealed no difference between heterozygous or homozygous animals and WT littermates in terms of motor ability, appearance and behavior, survival (to 1 year), overall tissue morphology, muscle innervation or spinal cord histology. These results suggest that increased levels of sIL6R alone do not affect normal physiology.

### 2.6. SOD1^G93A^ Mice

Breeding pairs for the *SOD1^G93A^* [B6.Cg-Tg (SOD1*G93A) 1GurJ (004435)] mouse model were obtained from the Jackson Laboratory (Bar Harbor, ME, USA). Non-transgenic wild-type (WT) females and *SOD1^G93A^* males were bred to generate the *SOD1^G93A^* mice and non-transgenic WT littermates that were used in the experiments. Genotyping was performed with standard primers against mutant SOD1 [11,18,19]. Each genotype PCR reaction used 0.5 ng of DNA/sample. Each genotyping experiment included a water control, a positive SOD1 DNA sample that was typically from a male SOD1 mouse obtained from the Jackson Laboratory for our breeding colony and a non-transgenic/WT DNA sample that was typically from a female mouse for the breeding colony. Only animals whose PCR products for SOD1 exhibited characteristics of the positive and negative control products were used in experiments.

### 2.7. SOD1^G93A^ X Il6ra Mouse Crosses

To generate sufficient numbers of animals together with non-transgenic (WT), non-*SOD1^G93A^* X *Il6ra* age- and sex-matched littermate controls to attain sufficient power as determined in our previous studies and prescribed for preclinical studies [19,20,21,22,23,24,25] we established the following breeding pairs:

*Homozygous* (*Il6ra+/+*), heterozygous *(Il6ra+/−*) and wild-type (*Il6ra−/−*) females of the same litter from the *Il6ra* colony were crossed with a *SOD1^G93A^* X *Il6ra+/−* male. Offspring from each breeding pair were considered littermates, genotyped for *Il6ra* and *Sod* variants and assigned to experimental groups as appropriate. To ensure that animals with a consistent copy number and phenotypes were included to the fullest extent possible, for all experiments sex and genotype littermate controls were used. To the fullest extent possible, experiments were blinded so that the individual performing the analysis did not know the genotype until the analysis was complete.

### 2.8. Animal Welfare and Behavior Analysis

To determine the onset of overt motor deficits as determined by the failure to fully extend the hindlimbs for greater than two seconds, SOD1 animals were monitored every other day beginning at postnatal day (P) 50. The first day when the animals failed to reach this milestone was recorded. In animals treated with the functional blocking antibody to IL6ra, a paw grip endurance (PaGE) [26] test was also performed and monitored once a week beginning at week 5. Animals were determined to reach the end-stage when they failed to right themselves within 10 s of being placed on their sides [27].

### 2.9. Therapeutic Intervention

To inhibit IL6 signaling, mice were treated with a commercially available antibody to mouse IL6R to determine if inhibition of the IL6R receptor (as occurs in patients treated with tocilizumab, see [8]) delayed development of overt behavior deficits and improved motor behavior. InVivoMAb anti-mouse Il6ra15A7 monoclonal antibody reacts with the mouse IL6 receptor and inhibits IL6 from binding (manufacturer’s data, BioXcell, Lebanon, NH, USA, #BE0047) [28]. Tocilizumab has been shown to not block IL6 signaling in mouse cells [29]. In these experiments, independent groups of littermate controls were administered the antibody, the isotype control (ant-keyhole limpet hemocyanin; BioXcell, # BE0090) or vehicle (InVivoPure Dilution Buffer, BioXcell, # IP0070) twice/week beginning at P30. The dose mirrored that used in the tocilizumab trial (8 mg/kg; approximately 160 μg/mouse) but was administered intraperitoneally, as i.p. in mouse has an equivalent delivery to intravenous administration [30] but with less tissue damage at the injection site. ELISA assays to determine serum sIL6R levels were used to confirm the drug distribution.

### 2.10. Histological Approaches to Determine NMJ Innervation, MN Counts and Glial Activation

All histological approaches were followed as previously described [23,24,25]. Following deep anesthesia, the animals were transcardially perfused with 2% paraformaldehyde in 0.1 mol/L sodium phosphate buffer. The animals were post-fixed at 4 °C for 2 h, after which the muscles were dissected, washed in phosphate-buffered saline and placed in two 24 h changes of 10% sucrose in phosphate buffer, followed by two 24 h changes of 20% sucrose in phosphate buffer, followed by being embedded in OCT and stored at −80 °C until sectioned. The spinal column was also dissected and placed into fixative for 24–48 h at 4 °C. Spinal cords were then dissected and processed as described for the muscles above. Continuous series of five, 20 μm thick consecutive cryostat sections were collected through the L2-3 regions of the spinal cord and mounted onto microscope slides that were stored at 4 °C. Each series of consecutive slides was then processed for MN counts or the determination of glial activation as described below.

### 2.11. Motoneuron Counts

One series of spinal cord sections were stained with a 0.1% cresyl violet solution (Abcam AB246816, Waltham, MA, USA) as previously described [31]. Only healthy MNs were counted at every 300 μm of the L2-3 regions, using a well-established reliable method that has been validated against an optical fractionator unbiased stereological counting method [32]. Healthy MNs are those that lie completely in the section with a nucleolus and normal MN morphology. Alternately, an adjacent series of spinal cord sections were processed for immunohistochemistry for choline acetyl transferase (ChAT) to identify MNs (goat anti-ChAT, Millipore AB144P), followed by incubation with secondary antibody (1:500, Alexa Fluor donkey anti-goat IgG; Invitrogen #A21206 (488) or A31572 (555). Fluorogel containing DAPI (Electron Microscopy Sciences, Hatfield, PA, USA) was used to mount coverslips to the glass slides. We confirmed that in animals where both approaches were used, the numbers of MNs determined were the same. Only one approach/animal was used for the reported analysis. The number of MNs/section were counted and the average/animal determined. Results are expressed as means and SEM for each group.

### 2.12. Glial Cells

Glial cells were identified as previously described [23]. Briefly, adjacent tissue sections of the spinal cord were processed for immunohistochemistry for ionized calcium-binding adapter molecule 1 (rabbit anti-Iba1; Wako 019-19741, 1:500) and CD68 (rat anti-CD68; BioRad MCA1957, 1:50) to identify microglial cells. After a series of washes, appropriate Alexa Fluor secondary antibodies (1:500, Alexa Fluor donkey anti-rabbit or Alexa Fluor donkey anti-rat; Invitrogen #A21208 (488 anti-rat) or #A48270 (555 anti-rat) were applied. Alternate sections were processed for immunohistochemistry for glial fibrillary acidic protein (rabbit anti-GFAP, Cell Signaling P14136, 1:500). Sections were incubated overnight at room temperature with primary antibodies, followed by species-specific Alexa Fluor-conjugated secondary antibodies (1:500, Invitrogen). After staining, the slides were mounted with Fluorogel containing DAPI (Electron Microscopy Sciences, Hatfield, PA, USA).

To assess glial cell activation, the fluorescence pixel area was measured using ImageJ 1.53 software (Frederick, MD, USA). Imaging was performed on either the left or right ventral horn of the lumbar spinal cord, with one side used for each animal in sections of every 300 μm of the L2-3 regions of the spinal cord. Representative photomicrographs, encompassing an area of 430 μm °ø 650 μm, were taken, using an Olympus BX51 fluorescent microscope (Olympus, Center Valley, PA, USA) with an Optomrics Microfire microscope camera (Optomrics, Tulsa, OK, USA, model S97808) and the Neurolucida software (64 bit, MBF Bioscience, Williston, VT, USA) of each analyzed section. The lumbar lateral ventral horn was manually outlined based on anatomical landmarks visible in the tissue sections. Manual thresholding was applied to the entire outlined image by systematically reducing the area highlighted until background staining was excluded and only positively stained activated glia (Iba1 or CD68) or astrocytes (GFAP) remained highlighted. The percentage area of positive staining was calculated for each section, and the average percentage area across all sections was determined for each mouse. Mouse-level averages were used to calculate the mean immunofluorescence for each group.

### 2.13. NMJ Innervation

For counting innervated hindlimb skeletal muscle NMJs, an immunohistochemistry was performed on soleus and tibialis anterior (TA) muscles as described previously [19,23,24,33]. The muscles were embedded in OCT and cut at 35 μm on the cryostat. To identify axons and presynaptic terminals, the sections were processed for immunohistochemistry for neurofilament light chain (1:1000, rabbit anti-NF-L; Novus NB300-135) and vesicular acetylcholine transporter (1:400, rabbit anti-VAChT; Invitrogen PA5-77386). After extensive washing in PBS, appropriate Alexa-fluor donkey anti-rabbit secondary antibodies (1:500, Invitrogen) were used for the detection of the primary antibodies. To identify postsynaptic terminals, sections were also labeled with Alexa-fluor–alpha-bungarotoxin (1:500 Invitrogen B13422 (488) or B13422 (555) [34]. NMJs that exhibited an overlap of red and green were considered innervated, while those that exhibited only α -BTX expression were considered denervated. All NMJs in every 30 μm section were analyzed. The percentage of innervated NMJs was determined in each treatment group using previously established counting criteria [23]. Tissue sections from both WT and *SOD1^G93A^* mice were processed simultaneously during the immunohistochemistry.

### 2.14. Statistical Analysis

Significant differences between the groups of mice (e.g., male vs. female) were analyzed using Student’s unpaired *t*-test. For all the experiments, we did not detect differences between males and females. While we maintained equal numbers of males and females for all groups, they were combined for the results presented here. Multiple groups were compared using analysis of variance (ANOVA) with Bonferroni’s *post hoc* pairwise analysis. Differences were considered statistically significant at *p* ≤ 0.05. All results are expressed as the mean ± standard error of the mean (SEM).

### 2.15. Spatial Transcriptomic Analysis

The 10x Genomics Visium Spatial Gene Expression platform was used to analyze the transcriptome in the spinal cord sections. *SOD1^G93A^* and *SOD1^G93A^* X *Il6ra^TMD^* mice were deeply anesthetized and decapitated and the spinal cords rapidly dissected and embedded in Tissue-Tek OCT, frozen and stored at −80 °C. Cryosections cut at 10 μm thickness were used for analysis. Tissue optimization (10X Genomics, Pleasanton, CA, USA, cat# 1000193) was performed according to the manufacturer’s guidelines and a 15 min permeabilization time was selected. Sections of the lumbar spinal cord were mounted on Visium Spatial Gene Expression Slides (10X Genomics, Pleasanton, CA, USA, Cat# 1000187) and stored overnight at −80 °C. For analysis, the slides were fixed, stained with hematoxylin and eosin (H&E), imaged using a Keyence BZ-X700 microscope and permeabilized for mRNA poly(T) sequence capture onto the underlying 55 μm diameter barcoded spot array on the slide surface (~5000 spots per 6.5 mm^2^ analysis box area). cDNA was generated and used for library construction. Indexed libraries were pooled for paired-end sequencing on an Illumina NovaSeq 6000, targeting > 75,000 reads per tissue-covered spot (coverage area estimated from captured images). Sections adjacent to the gene expression sample (before and after) were collected on slides and processed using immunohistochemistry for ChAT, IBA and GFAP to identify MNs, microglia and astrocytes, respectively, as described above.

Data analysis was performed using the 10X Genomics Space Ranger suite of analysis tools. Raw .bcl were demultiplexed using the spaceranger mkfastq tool. The spaceranger count, spaceranger aggr and spaceranger reanalyze tools were used for aligning and filtering fastq files and normalizing and performing the dimensionality reduction and clustering of gene expression data. RNA reads were aligned to the mouse GRCm38/mm10 reference genome assembly. A single spot data visualization was performed using the Loupe-Cell-Browser 2.0.0. The differential expressed genes (DEGs) were functionally evaluated by using Ingenuity Pathway Analysis (IPA).

In this study, an analysis was performed on six comparable sections of the L2-3 spinal cord from one female *SOD1^G93A^* and one female *SOD1^G93A^* X *Il6ra^TMD^* mouse at P80. A total of 8 MN-enriched spots/animal were identified by the expression of mnx (HB9), together with H&E staining to confirm the presence of MNs. Surrounding areas to those where MNs were located that were positive for GFAP and/or Iba1 expression were analyzed as glial cell-enriched. A total of 28 *SOD1^G93A^* and 36 *SOD1^G93A^* X *Il6ra^TMD^* glial areas were analyzed.

## 3. Results

**Figure 2:**
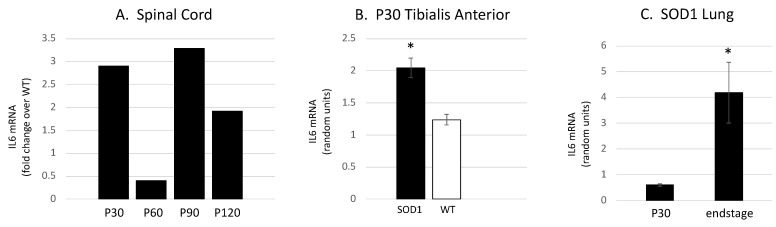
IL6 mRNA levels are increased in tissues involved in ALS pathology in the *SOD1^G93A^* mouse. (**A**) To determine if IL6 expression levels change in the ventral lumbar spinal cord with disease progression, we performed a preliminary rtPCR experiment. Message levels for cytokine are increased in SOD1 mice as compared to WT at P30, 90 and 120. Interestingly, the pattern mirrors the microglial activation previously observed [23]. (**B**) *Il6ra* mRNA is expressed at higher levels in the SOD1 mouse TA muscle as compared to wild-type age-matched controls at P30, coincident with the initial NMJ denervation previously observed [24] (* *p* < 0.001; Student’s *t*-test; n = 6 per group). (**C**) Relative to age-matched wild-type controls, *Il6ra* mRNA is expressed at higher levels in the SOD1 lung at end-stage (p120) than at p30 (* *p* = 0.028; Student’s *t*-test; n = 4 per group).

To begin to determine the pathological significance of IL6 trans-signaling in ALS progression, we initially determined the expression of IL6 in the *SOD1^G93A^* mouse tibialis anterior muscle (TA), lumbar spinal cord and lung coincident with the NMJ denervation, glial activation and MN degeneration previously characterized [19,23,24,33,35]. We identified increased IL6 expression in the TA muscle coincident with early NMJ denervation, increased expression in the spinal cord that appears to parallel microglial activation and increased expression in lung tissue at end-stage (Figure 2A–C).

### 3.1. SOD1 X Il6ra Mice Exhibit Earlier Symptom Onset

**Figure 3:**
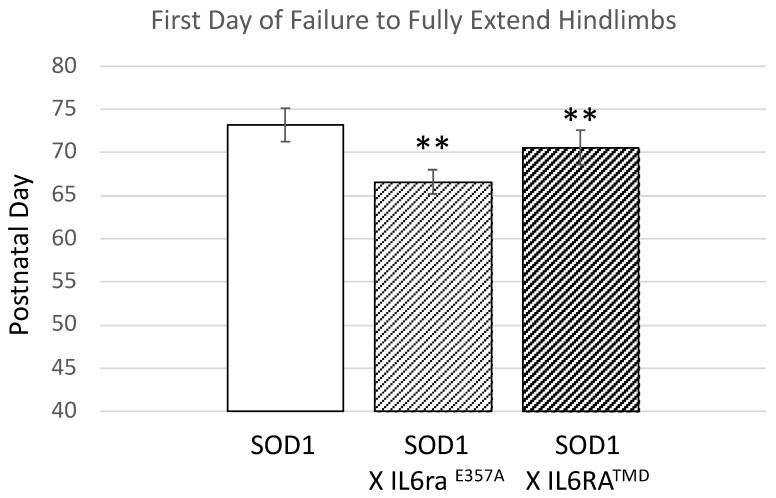
Overt motor deficits appear earlier in ALS *SOD1^G93A^* mice with enhanced IL6 trans-signaling. Animals were monitored three times/week beginning at postnatal day 50. The SOD1 X *Il6ra^E357A^* and SOD1 X *Il6ra^TMD^* mice failed to fully extend their hindlimbs for greater than two seconds earlier than SOD1 animals. Shown is the first day when animals failing to reach this milestone was recorded (mean + SEM, SOD, n = 28; 8F; 20 M; SOD X *Il6ra^E357A^*, n = 74; 33F, 41 M, 19 hm, 55 ht; SOD1 X *Il6ra^TMD^*, n = 35, 11 F, 24 M, 11 hm, 24 ht). Statistical analysis was performed using one-way ANOVA followed by Bonferoni post hoc test. ** *p* < 0.01. No differences within genotypes between male and female or between heterozygous or homozygous were detected.

We crossed both *Il6ra* mouse models with the *SOD1^G93A^* mouse model. Serum sIL6R levels corresponded to levels observed in the Il6ra genotypes. We routinely began monitoring the *SOD1^G93A^* mice for overt disease deficits and welfare beginning at postnatal day (P) 50. Using failure to exhibit full leg extension as the onset of overt motor deficits, e.g., [19], the *SOD1^G93A^* X *Il6ra^E357A^* and *SOD1^G93A^* X *Il6ra^TMD^* mice had a significantly earlier onset as compared to *SOD1^G93A^* (Figure 3). Within each group, there were no differences in onset between the sexes (*SOD1^G93A^*; *SOD1^G93A^* X *Il6ra^E357A^*; or *SOD1^G93A^* X *Il6ra^TMD^*). There were no statistical differences between heterozygous and homozygous within the Il6ra crosses (*SOD1^G93A^* X *Il6ra*^E357A^; or *SOD1^G93A^* X *Il6ra^TMD^*), nor between the *SOD1^G93A^* X *IL6ra^E357A^* and *SOD1^G93A^* X *IL6ra^TMD^* crosses. For all subsequent studies, only homozygous *IL6ra^E357A^* and *IL6ra^TMD^* were used.

### 3.2. Enhanced IL6 Trans-Signaling Appears to Enhance NMJ Denervation in Resistant NMJs

**Figure 4:**
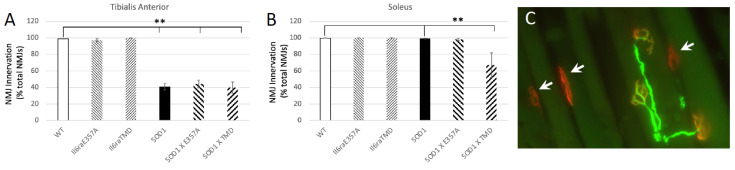
(**A**,**B**) Muscle innervation was examined in the P80 TA and soleus in software in wild-type (WT; n = 12, 6F, 6 M), *Il6ra^E357A+/+^* (n = 11, 6 M, 5F), *Il6ra^TMD^* (n = 9; 4 M, 5F), SOD1 (n = 12; 7 M, 5F), SOD1 X *Il6ra^E357A+/+^* (n = 8, 3 M, 5F) and SOD1 X *Il6ra^TMD+/+^* mice (n = 8, 4 M, 4F). (**C**) Shown is a representative photomicrograph of NMJs in the TA muscle of a *SOD1^G93A^* mouse. Post-synaptic terminals are identified with α-BTX (red) and pre-synaptic terminals and axons identified with anti-bodies to VAChT and NF (green). The arrows indicate denervated NMJs as there is no overlap with VAChT. While significant denervation occurs in the TA in SOD1 by P80, we did not observe differences between SOD1 and SOD1 animals with enhanced IL6 trans-signaling. At P80, significant denervation is occurring in the SOD1 mouse where all IL6 signaling occurs via trans-signaling. NMJ innervation was performed as previously described [23,24,25]. The results are presented as the % denervated of total NMJs/muscle (mean + SEM). ** *p* < 0.01 vs. WT or SOD1 as determined by one-way ANOVA followed by Bonferroni and Holm post hoc tests.

We examined neuromuscular junction innervation/denervation in animals from the cohort above. There were no differences in innervation patterns in the muscles of WT or enhanced trans-signaling mice. By P80, the denervation of NMJs is well underway in the TA muscle, as previously reported [24,25], and we did not observe differences between SOD1 and SOD1 animals with enhanced trans-signaling (Figure 4). Interestingly, in the SOD1 X *Il6ra^TMD^* model of enhanced trans-signaling, we observed a statistically significant denervation of the soleus muscle at P80, whereas, consistent with previous results [24,25], we did not detect denervation of the soleus muscle in SOD1 mice.

### 3.3. Enhanced IL6 Trans-Signaling Results in Increased Astrocyte and Microglia Activation but No Change in Motoneuron Survival at Postnatal Day 80

**Figure 5:**
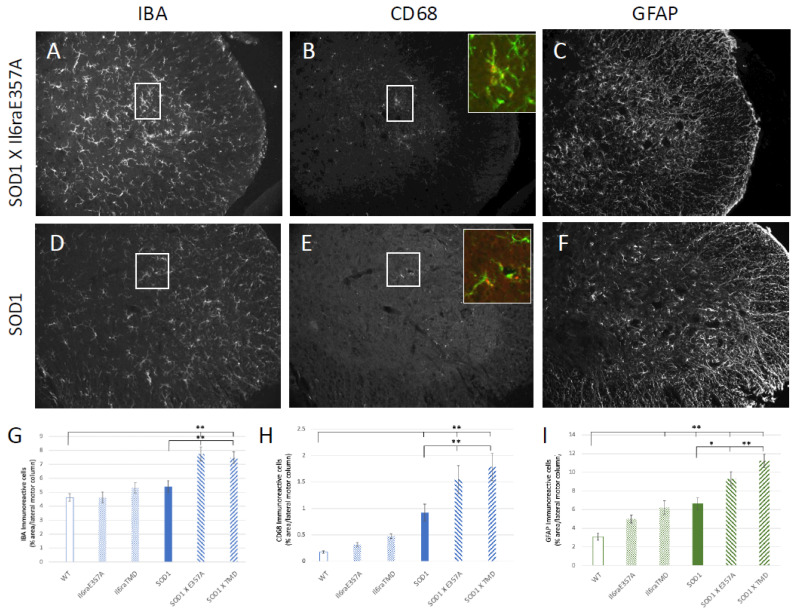
Representative images are shown of immunohistochemical staining of microglia with IBA and CD68 of microglia in P80 L3-4 lateral motor column of SOD1 and SOD1 X *Il6ra^E357A^*^+/+^ mice (**A**,**B**,**D**,**E**). Representative images are shown of immunohistochemical staining with GFAP of astrocytes of the same animals (**C**,**F**). Quantification of percent area of IBA (**G**), CD68 (**H**) or GFAP (**I**) was performed using Image J software in wild-type (WT; n = 8, 4F, 4 M), *Il6ra^E357A+/+^* (n = 9, 5 M, 4F), *Il6ra^TMD^* (n = 10; 5 M, 5F), SOD1 (n = 11; 7 M, 4F), SOD1x *Il6ra^E357A+/+^* (n = 8, 4 M, 4F) and SOD1 X *Il6ra^TMD+/+^* mice (n = 8, 4 M, 4F). Adjacent sections were processed for GFAP and IBA/CD68, so data shown in A/B and DE are from same animals. * *p* < 0.05; ** *p* < 0.01 vs. WT or SOD1 vs. SOD1 X *Il6ra* models as determined by one-way ANOVA followed by Bonferroni and Holm post hoc tests.

To begin to determine differences in pathological events that may account for the earlier symptom onset, we also focused on glial activation, as increases in CSF IL6 were unique to ALS patients vs. healthy or disease controls [5], and in the clinical trial treating ALS patients with the IL6R inhibitor tocilizumab (TCZ), only individuals inheriting the Ala^358^ variant and treated with TCZ showed decreases in CSF c-reactive protein (CRP), a marker of inflammation [8]. We examined “glial activation” as determined by an increased expression of GFAP for astrocytes. For microglial activation, we determined IBA1 expression together with CD68 (ED1), as we have found this to remain the most reliable measure, immunohistochemically, of activated microglia [36,37]. There was an increased expression of all glial markers in SOD1 X *Il6ra*^E357A/E357A^ and SOD1 X *Il6ra*^TMD/TMD^ as compared to SOD1 at P80 (Figure 5). Microglial “activation”, as determined by an increased expression of IBA and CD68, was increased in SOD/trans-signaling crosses (SOD1 X *Il6ra^E357A^*; SOD1 X *Il6ra^TMD^*) as compared to SOD1 animals. Astrocyte activation, as determined by an increased expression of GFAP, was increased in SOD/trans-signaling crosses (SOD1 X *Il6ra^E357A^*; SOD1 X *Il6ra^TMD^*) as compared to SOD1 animals, indicating that IL6 trans-signaling is an important factor in astrocyte activation. Interestingly, although reduced when compared to their SOD1 counterparts, the *Il6ra^E357A/E357A^* and *Il6ra^TMD/TMD^* mice showed an increased immunohistochemistry for GFAP (astrocytes) as compared to WT animals, suggesting, as previously shown [1], that glial cells may be primed by IL6R trans-signaling even with physiological levels of IL6.

We did not detect any differences in the number of surviving motoneurons between the SOD1 and SOD1 X *Il6ra* models at P80.

**Figure 6:**
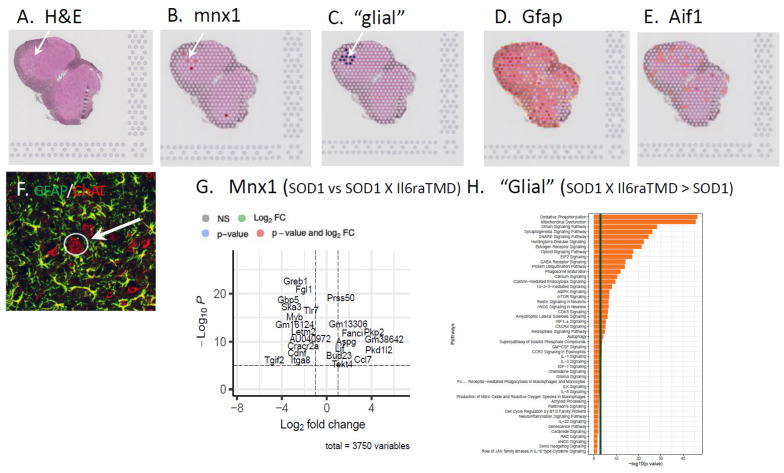
To begin to investigate if there is a specific, IL6 trans-signaling-mediated glial response and if spinal MNs have unique responses in conditions of trans-signaling, we used a discovery-level whole transcriptome-based spatial RNAseq analysis of the ventral, lateral lumbar spinal cord of SOD1 and SOD1 X *Il6ra*^TMD^ mice. The overall cytoarchitecture and cells exhibiting MN phenotypes were identified by H&E staining and the image capture of sections prior to RNA isolation (**A**). (**B**) Individual 55 um diameter analysis spots containing MNs were identified by the expression of the mnx1 (HB9; [38]) and mnx1-negative, GFAP and/or Aif1 (IBA1) surrounding glial spots selected (**C**–**E**). (**F**) Photomicrograph of an adjacent spinal cord section processed by double-label immunofluorescence for ChAT to identify MNs (red) and GFAP to identify astrocytes (green) in the same MN-enriched region as shown in (**A**) (arrows in (**A**,**F**)). (**G**) The enhanced volcano plot indicates the extent of differential gene expression between MNs of SOD1 vs. SOD1 X *Il6ra^E357A^* mice. (**H**) Pathway analysis was performed and identified IL6 trans-signaling-specific pathways associated with glial responses at P80 in SOD1 X *Il6ra*^TMD^ mice.

Our data suggest that in the SOD1 mouse model of ALS, enhanced IL6 trans-signaling promotes greater microglial and astrocyte activation (Figure 5). In the TCZ study, patients inheriting the *Il6R* Ala^358^ variant exhibited reduced CSF CRP levels following treatment with the antibody, whereas those without the allele showed no change in CSF CRP [8]. To begin to investigate if there is a specific, IL6 trans-signaling-mediated glial response and if spinal MNs have unique responses in conditions of trans-signaling, we used a non-biased spatial RNAseq analysis of the ventral, lateral lumbar spinal cord of SOD1 and SOD1 X *Il6ra*^TMD^ mice. 10x Genomics Visium Spatial transcriptomics reveals discovery-level gene expression architecture in native tissue. This powerful approach allows a pathway analysis targeting a 1–10 cell resolution within the spatial confines of the tissue architecture. Individual 55 μm in diameter spots containing MNs were identified by the expression of mnx1 (HB9, [38]; Figure 6A,B,F). The enhanced volcano plot indicates the extent of differential gene expression between MN-enriched spots between SOD1 vs. SOD1 X *Il6ra^E357A^* mice (Figure 6G). For every mnx1 spot identified, the surrounding spots that were mnx1-negative and AIF1- and/or GFAP-positive were identified as “glial”-enriched and analyzed (Figure 6C,D,E). A pathway analysis was performed, and it identified IL6 trans-signaling-specific pathways associated with glial responses at P80 in SOD1 X *Il6ra*^TMD^ mice (Figure 6H). Although cost limitations negated a deeper spatial analysis and larger sample size, the results of these experiments indicate that enhanced IL6 trans-signaling resulted in differential gene expression in both MNs and glial cells, with a strong impact on glial metabolism that warrants further investigation.

### 3.4. Treatment with IL6R Blocking Antibody Had No Effect on Symptom Onset or Survival

**Figure 7:**
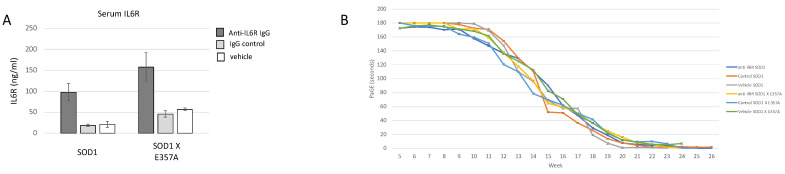
To inhibit IL6 signaling, mice were treated with a commercially available antibody to mouse IL6R (anti-IL6R 15A7) to determine if development of overt behavior deficits was delayed and motor behavior improved. (**A**) The 15A7-treated mice exhibited increased serum sIL6R as compared to the control of vehicle-treated animals (SOD1: 15A7-treated n = 34, isotype control n = 4, vehicle n = 2; SOD1 X *Il6ra^E357A^*: 15A7-treated n = 4, isotype control n = 3, vehicle n = 3). Results are expressed as the mean ± standard error of the mean (SEM). (**B**) There were no differences in the decline in performing the paw grip endurance test between the treated groups (SOD1: 15A7-treated n = 10, 5F, 5 M; isotype control n = 9, 5 M, 4F; vehicle n = 9, 5 M, 5F; SOD1 X *Il6ra^E357A^*: 15A7-treated n = 11, 5 M, 6F, isotype control n = 9, 4 M, 5F, vehicle n = 9, 4 M, 5F). Shown are the MEAN PAGE results + standard error of the mean (SEM). The slope of decline was calculated for each animal, and significant differences between the groups of mice were compared using a one-way analysis of variance (ANOVA) with Bonferroni’s *post-hoc* pairwise analysis. All results are expressed as the mean ± standard error of the mean (SEM).

To inhibit IL6 signaling, mice were treated with a commercially available antibody to mouse IL6R (anti-IL6R 15A7) to determine if inhibition of the IL6R receptor delayed development of an overt behavior deficit and improved motor behavior. The 15A7-treated mice exhibited a 2–3-fold increase in serum sIL6R (Figure 7A), similar to that observed in ALS patients treated with tocilizumab [8]. There were no differences in overt motor symptom onset, paw grip endurance (Figure 7B) or survival, between the 15A7 antibody, isotype control antibody or vehicle-treated SOD1 or between similar treatments in SOD1 X *Il6ra^E357A^* mice. Because we did not observe any effect of the treatment across the phenotypes and the extensive breeding necessary to generate these mice, we suspended the study with a reduced number of animals/group as recommended for preclinical trials [20,21,22].

## 4. Discussion

While several inflammatory cytokines are reported to have an elevated expression in ALS, interleukin 6 (IL6) is an intriguing therapeutic target and a key focal point of mechanistic and clinical ALS research. IL6 is a multi-functional cytokine that influences diverse cellular mechanisms [10,39,40,41,42]. In subjects with ALS, IL6 levels are elevated in the skin, serum and CSF [5,6,43,44,45,46]. Of relevance to MNs, IL6 is a member of the CNTF/CT/LIF gp130 family of trophic factors that are critical in MN development and survival following injury and disease [47,48,49]. Accordingly, IL6 signaling may be beneficial in maintaining neuromuscular junction (NMJ) innervation and promoting MN survival. Indeed, in conditions of axonal injury, muscle injury, muscle stress (e.g., exercise) and atrophy (e.g., sarcopenia), increases in the muscle expression of IL6 are correlated with increased levels of circulating IL6 [50,51,52].

To better understand how IL6 trans-signaling may modify disease progress in ALS, we created mouse models where the trans-signaling is enhanced in the presence of IL6 because the concentrations of soluble IL6R are substantially greater than those of wild-type animals. The Il6rE357A mouse model is a useful experimental model to investigate trans-signaling as it occurs in patients who inherit the IL6R Asp358Ala allele, as this model exhibits comparable levels of circulating IL6R as observed in previous clinical studies [53] (see Figure 1). The *Il6ra^TMD^* mouse provides a pure experimental model, as the deletion of the transmembrane domain of IL6R results in profound increases in circulating soluble receptor, and in the homozygous mice all IL6 signaling is predicted to occur via trans-signaling. A similar approach has been taken to investigate liver regeneration [54]. The models may prove to be useful for studies of neurodegenerative disorders and other non-CNS disorders and will be deposited with the Jackson Laboratory.

Initial muscle denervation occurs in the TA muscle (fast-fatigable and fatigue-resistant fibers) between P14 and 30, while little denervation occurs in the soleus muscle (slow and fatigue-resistant fibers) even late in ALS progression [24,25]. Interestingly, while glial activation is observed shortly after the initial NMJ denervation, robust microglia and astrocyte activation is not observed until later in ALS progression e.g., see [23,24,25,33]. In this study, we identified increased IL6 expression in the TA muscle coincident with early NMJ denervation, increased expression in the spinal cord that appears to parallel microglial activation and increased expression in lung tissue at end-stage in the *SOD1^G93A^* mouse model (see Figure 2).

When crossed with the SOD1 mouse model, there did not appear to be differences in levels of circulating cytokine levels in the SOD1 or SOD1 X *Il6ra* models, suggesting that differences in pathology are likely due to IL6 trans-signaling-mediated cellular pathophysiology rather than an increased availability of the cytokine. To begin to investigate potential pathology-associated mechanisms that may be modulating disease progression, our examination was focused on a single time point coincident with overt motor deficits, i.e., the failure to extend the hindlimbs. While we did not detect differences in the extent of denervation in the TA muscle that typically undergoes NMJ denervation, we did see denervation in the soleus muscle of the SOD1 X *Il6ra^TMD^* mice where all IL6 signaling occurs via trans-signaling (see Figure 4). In the SOD1 model, this muscle does not typically undergo NMJ denervation (see [24,25]). Interestingly, there were no changes in MN appearance or physical degeneration, as there were no differences in the numbers of MNs in the lumbar spinal cords of the SOD1 or SOD1 X *Il6ra* models. While IL6 signaling may have beneficial effects as a trophic factor, our results suggest that this function is likely thwarted by IL6′s other potential roles, such as in the coordination of the inflammatory and immune responses. Indeed, in ALS patients who inherit the *Il6R* Ala^358^ allele, IL6 levels are correlated with decreases in muscle strength and decreased lung function [6].

In the tocilizumab (Actemra^®^) clinical trial in ALS “clinicaltrials.gov number NCT02469896 (accessed on 8 January 2025)”, an unexpected (not a predefined study outcome biomarker), but nonetheless interesting, result was that the reduction in CSF C-reactive protein (CRP) occurred only in the CSF of treated ALS patients with at least one *Il6R* Ala^358^ allele [8]. We believe these results suggest IL6 trans-signaling mediates distinct CNS glial responses in individuals inheriting the *Il6R* Ala^358^ allele. IL6 is associated with microglia and astrocyte activation, which have been shown to promote MN dysfunction and degeneration [1,55,56,57,58]. Our results indicating profound glial cell activation in the SOD1 X *Il6ra* models suggest enhanced trans-signaling may have an influence on this response (see Figure 5). To initially identify how IL6 trans-signaling may modulate the cellular physiology, we performed a spatial transcriptomic analysis of SOD1 and SOD1 X *Il6ra^TMD^* L2-3 spinal cord sections. While the results are limited, they did readily indicate differences in MN gene expression and potential glial cellular pathway activation between the animals with and without enhanced trans-signaling (Figure 6). As to whether this represents distinct differences in cellular pathophysiology or changes in the timing of the events that may be associated with disease progression will have to be the focus of future investigations.

Interestingly, while there was an earlier onset of failure to extend the hindlimbs (Figure 3), there were no differences in overall survival between the SOD1 and SOD1 X *Il6ra* models. Here the mouse models recapitulated our results in ALS patients, where those inheriting the *Il6R* Ala^358^ allele exhibited faster disease progression early in the disease, while there were no differences in overall survival [5]. In individuals where there is enhanced IL6 trans-signaling, individual aspects of the disease may be modified or worsened, such as increased NMJ denervation or an even faster decline in respiratory function, but the overall time to end-stage may not change. In this case, therapeutic strategies may be valuable for improving patient quality of life.

In the tocilizumab (Actemra^®^) clinical trial in ALS, tocilizumab was found to be safe but did not alter ALS progression. The absence of an effect on ALS progression was perhaps because of the short duration of the study and/or the selection of screening biomarkers. Increases in sIL6R were observed in all tocilizumab-treated patients, likely reflecting the altered catabolism of the sIL6R/IL6/tocilizumab complexes as reported in other patient populations treated with the drug [59]. We observed similar results in the mouse models, where treatment with a mouse equivalent antibody to IL6R failed to affect the onset of overt motor deficits and overall survival (see Figure 7). Interestingly, increases in soluble IL6R were also observed in mice treated with antibody. While it is tempting to conclude that these treatment approaches are not beneficial, it remains unknown, and indeed unlikely, that the functional blocking antibodies are reaching the target areas in the spinal cord or cortical parenchyma in sufficient concentrations to be effective (see [60,61,62,63]).

## 5. Conclusions and Study Limitations

To better understand how IL6 trans-signaling may modify disease progress in ALS, we created mouse models where the trans-signaling is enhanced in the presence of IL6 because the concentrations of soluble IL6R are substantially greater than those of wild-type animals. When crossed with the SOD1 mouse model of ALS, we observed that in animals with enhanced trans-signaling, symptom onset and the pathological processes were accelerated, suggesting a role in disease modification. Administration of the IL6R with a functional blocking antibody had no effect.

Our study is an initial survey of the potential pathological consequences of enhanced IL6 trans-signaling in ALS disease progression. It is limited in that the examination of these events was initially determined at a single time point, and the spatial transcriptomics analysis could only confirm differences between two animals. Future work to investigate the site-specific influence of enhanced IL6 trans-signaling and the tissue-specific bioavailability of potential therapeutics will be necessary to identify targets for precise therapeutic interventions that may limit disease progression in the 60% of ALS patients who inherit the common *Il6R* Asp^358^Ala variant.

## Data Availability

The raw data supporting the conclusions of this article will be made available by the authors on request.
